# Ultrafast Dynamics of Different Phase States Ge_2_Sb_2_Te_5_ Film Induced by a Femtosecond Laser Pulse Irradiation

**DOI:** 10.3390/ma15196760

**Published:** 2022-09-29

**Authors:** Hao Wu, Weina Han, Xiaobin Zhang

**Affiliations:** 1 Laser Micro/Nano-Fabrication Laboratory, School of Mechanical Engineering, Beijing Institute of Technology, Beijing 100081, China; 2 Beijing Institute of Technology Chongqing Innovation Center, Chongqing 401120, China

**Keywords:** GST, ultrafast dynamics, femtosecond laser excitation, phase state, phase transition

## Abstract

A femtosecond laser could realize a high transition rate of the phase change material (PCM), and the properties of the amorphous and the crystalline Ge_2_Sb_2_Te_5_ (GST) induced by a femtosecond laser were studied, which was one of the candidates among the PCMs. However, the characteristics of the intermediate phase states in reversible phase transitions were also important and helpful to explore the mechanisms of the phase transitions. In this paper, the ultrafast dynamics of amorphous, crystalline face-centered-cubic (FCC), and hexagonal-close-packed (HCP) states were investigated using a femtosecond laser pulse excitation through a reflective-type pump–probe technique, obtained by annealing at certain temperatures, and verified using X-ray diffraction (XRD) and the Raman spectrum. It was found that as the annealing temperature increased, the electron of the GST films could be excited more easily, while the ablation threshold decreased. Due to annealing, the structure of bonding was changed for different phase states, which resulted in the decrease in the band gap of the films. In addition, it was hard for the intermediate state films to transit to the amorphous structure state via the femtosecond laser, and the crystallization would be enhanced, while the crystalline HCP structures of GST could be directly and easily changed to the amorphous state by a pulse, which resulted from the non-thermal phase change caused by the excited electron.

## 1. Introduction

Phase change material (PCM), especially the ternary Ge-Sb-Te compounds (e.g., Ge_2_Sb_2_Te_5_) [1,2,3], has shown great potential in the applications of data storage [4,5], photonic switches [6,7], sensors [8], and so on. Its principle of working is according to the reversible phase transitions among the amorphous, the crystalline, and the intermediate phase states [9], which result in the changing of electrical optical refractivity, resistance, etc. [10]; the transformations can reach up to 10^12^ cycles [11], which are generally achieved by means of an electric field [12] or annealing for a certain time [13]. However, the surface of the materials could be destroyed by touching during the exertions of an electric field process, and the speed of the phase transition is slow. Actually, the amorphization process of GST typically could occur in tens of nanoseconds, and the crystallization process could happen in about tens to hundreds of nanoseconds [14]. Ultrafast laser irradiation, as a precise and controllable method, could solve the problems.

The microstructures [15,16,17], crystallization structure [18], reflectivity and gray-scale [19], etc., of the amorphous, crystalline, and intermediate phase states GST were investigated using the Raman spectrum [20], scanning electron microscopy (SEM) [16] and transmission electron microscopy (TEM) [17,21] and so on, which were characterized ex-situ. The results indicated the characteristics after irradiation by a femtosecond laser, and the phase transition mechanism of GST induced by a femtosecond laser was inferred. However, the interactions between the femtosecond laser and the materials were complicated. A pump–probe technique, as an in-situ observation method, was applied to explore this process. Siegel et al. [22] compared the amorphization of the crystalline GST by a femtosecond laser with that by a nanosecond laser using this technique, and the results showed that the femtosecond pulse was a more efficient method. Moreover, the dielectric function changed by 30% within 100 fs during the phase transitions [23]. The amorphous GST films induced by the femtosecond laser were also studied. Zhou et. al. [24] found that the crystallization rate was faster than the ablation rate of the GST film by the pump–probe technique, while the time resolution was more than 10 ps, which was too long to investigate the electron of GST excited by femtosecond laser. Zhang and Gan [25] found that the carriers could be excited within 1 ps and recovered less than 3 ns. In addition, the ultrafast dynamics of amorphous and quasi-amorphous GST films were compared in pump–probe experiments, and the mechanism of the crystallization enhancement effect was proposed [26]. However, there were the amorphous, crystalline, and intermediate phase states in reversible phase transitions, which had multiple applications e.g., non-volatile memory, because the characteristics of these phase states were different [27,28,29]. A few papers discussed the optical and electrical properties of different phase states [30], while the ultrafast dynamics needed to be studied, which was helpful to explore the mechanisms of the phase changes.

To fill this gap, the properties of three kinds of phase states GST films, e.g., amorphous, crystalline FCC, and HCP states are investigated in this paper. The different phase states GST are derived by annealing at certain temperatures, which are verified by XRD and Raman spectrum. Then, the ultrafast dynamics of these samples induced by a femtosecond laser pulse are studied by the reflective-type pump–probe experiments. In addition, the Raman spectrum results for the zones of three kinds of phase states GST films irradiated by a femtosecond laser pulse show the mechanism of phase changes between the amorphous and crystalline states. 

## 2. Experiment

**Figure 1:**
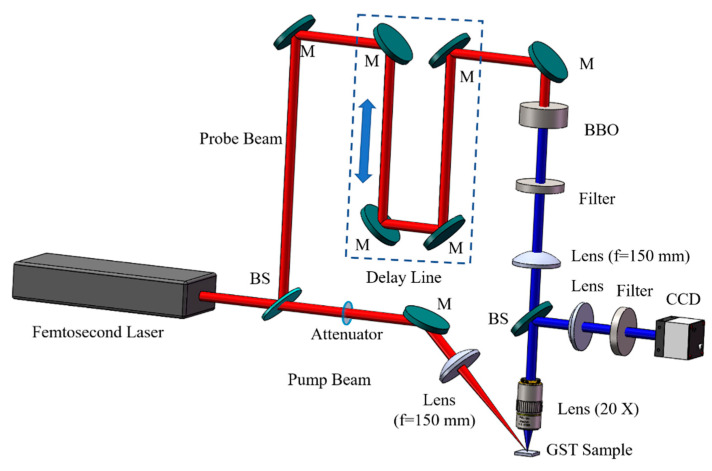
The schematic of the reflective-type pump–probe system (BS: beam splitter, M: mirror).

The GST films were deposited on a Si (100) substrate by a magnetron sputtering method from the Ge_2_Sb_2_Te_5_ target with Ar gas flow rate of 35 sccm at room temperature when the sputtering power was 30 W and the thickness was 100 nm, which was measured using a three-dimensional topography instrument. To study the ultrafast dynamics of amorphous, FCC, and HCP crystalline states, the as-grown GST films were annealed at a different temperatures from 150 °C to 350 °C for 30 min, and the different phase states were identified using XRD patterns and Raman spectrum. Then, the ultrafast dynamics of these films with different phase states were investigated using the reflective-type pump–probe shadowgraph imaging technique. As shown in Figure 1, a femtosecond laser (Spitfire, Spectra-Physics Inc., Mountain View, CA, USA) with a wavelength of 800 nm was controlled by a signal generator to emit laser pulses. Then, the generated laser beams were divided into pump and probe beams using a beam splitter (Thorlabs), and the intensity ratio between them was 0.45:0.55. The pump pulse was focused on the samples using a lens with a focal distance of 150 mm at an angle of 45°. The energy of the excitation beam was controlled using an attenuator. The other’s frequency was doubled using a beta barium borate (BBO) crystal. It was focused on the excitation area by the pump beam perpendicularly through a lens with a focal distance of 150 mm and a 20 × objective (NA = 0.45, Olympus Inc., Tokyo, Japan). The influence of the pump pulse was eliminated by a 400 nm bandpass filter. An optical delay line was used to make the probe beam delay to irradiate the excitation area with the pump beam and modify the time delay as precisely controlled using a computer. A charge-coupled device (CCD, Imaging Sources), whose parameters were set by the computer, was used to take reflected optical images. In addition, another 400 nm bandpass filter was used to eliminate the influence of the pump beam and the plasma generated by laser manufacturing of the samples in front of CCD. In the pump–probe experiments, the time delay started at 0 fs to study the ultrafast dynamics, which meant that the pump and probe beam irradiated the sample at the same time. The measured reflectivity was calculated by

(1) $$\Delta R/R_{0}=(R-R_{0})/R_{0}$$

where *R* and *R*_0_ are the intensities of a shadowgraph image at the measurement position with and without the pump beam irradiation, respectively. The reflective-type pump–probe experiments were carried out three times for different phase states GST films, and the average reflectivity of the area with 2 × 2 pixels for a measured position was selected to be the final value. Lastly, the zones on different phase states samples irradiated by laser beams were measured using a commercial Raman system (Nanophoton, Raman-11).

## 3. Results and Discussion

### 3.1. Different Phase States of Films at Different Annealing Temperatures

To confirm the chemical composition of the materials, the atomic ratio of the films was measured using EDS before the experiments. The films had a composition of Ge_22_._25_Sb_22_._32_Te_55_._43_, which is almost close to the desired stoichiometry.

**Figure 2:**
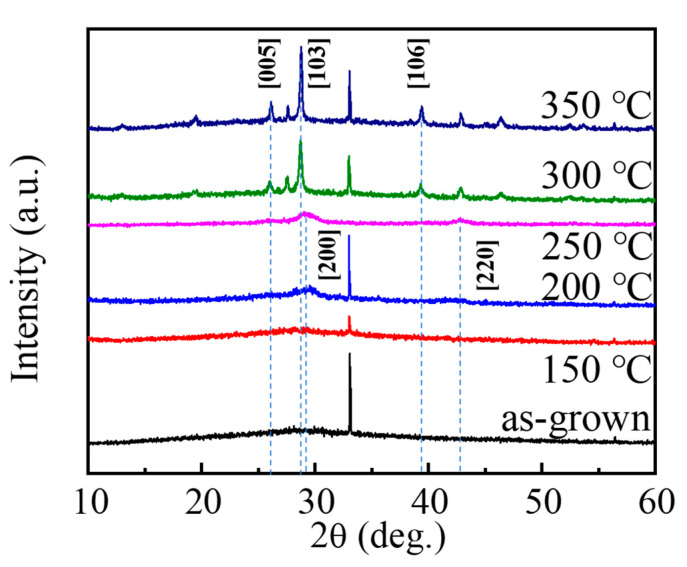
XRD patterns of the as-grown GST films annealed at different temperatures for 30 min.

Before studying the ultrafast dynamics of different phase states of GST, the as-grown films are annealed at various temperatures from 150 °C to 350 °C for 30 min, and then measured via XRD (Bruker, D8 focus), as shown in Figure 2. There are no peaks for the as-grown GST films and the films after annealed treatment at 150 °C, which means the films are in an amorphous phase. However, when the annealing temperature is at 200 °C for 30 min, the crystalline face-centered cubic (FCC) state with the peak of [200] emerges, which means that the phase transits from the amorphous state to the FCC structure. While the films are annealed at 250 °C for 30 min, there are peaks of [200] and [220] in the XRD patterns, which are also the characteristics of the FCC structure. As the annealing temperature increases to 300 °C and stays there for 30 min, the new peaks of [005], [103], and [106] are shown in Figure 2, and the HCP structure can be identified according to the peaks. When the annealing temperature continues growing and reaches 350 °C for 30 min, the peaks that emerge from the XRD spectrum are almost the same as those from annealing at 300 °C for 30 min, and the crystallization with hexagonal close packed (HCP) appears. 

**Figure 3:**
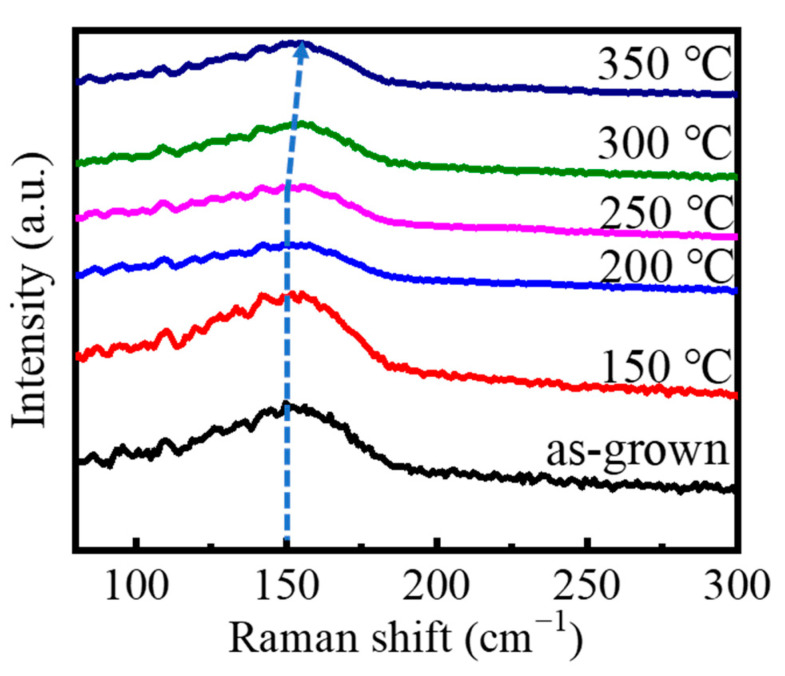
Raman spectrum of the as-grown GST films annealed at different temperatures for 30 min.

The samples annealed at different temperatures were also measured using the Raman system, as shown in Figure 3. According to the characteristic peaks of GST [26,29,31,32,33], it can be seen that a broad peak at about 150 cm^−1^ (Peak A) results from the stretching vibrations of Te-Te bonds Sb_m_Te_3_ (m = 1, 2) pyramidal units [31], and another peak at about 110 cm^−1^ (Peak B) is caused by the heteropolar bonds of GeTe_4_ or GeTe_3_Ge [26] for the as-grown GST and films annealed at 150 °C for 30 min. The relative intensity of Peak A to Peak B is about 1.4 for the amorphous GST, while it decreases to around 1.31 for the films annealed at 150 °C for 30 min. It continues decreasing from about 1.26 for the films annealed at 200 °C for 30 min to about 1.25 for those annealed at 250 °C for 30 min, which indicates that the crystalline FCC structure states are formed. It can be inferred that the bond of Ge-Te plays an important role when an amorphous state GST transits to the crystalline FCC structure. When the as-grown GST films are annealed at 300 °C and 350 °C for 30 min, there are peak shifts from Peak A to about 157 cm^−1^ (Peak C), which is due to the vibration of Sb-Sb bonds [32], which means that the phase state becomes the crystalline HCP structure. The results of the analysis of different phase states by the Raman spectrum are in agreement with those of XRD.

### 3.2. Spatial–Temporal Resolved Measurement of Femtosecond Laser Pulses Excited Different Phase States GST Films

**Figure 4:**
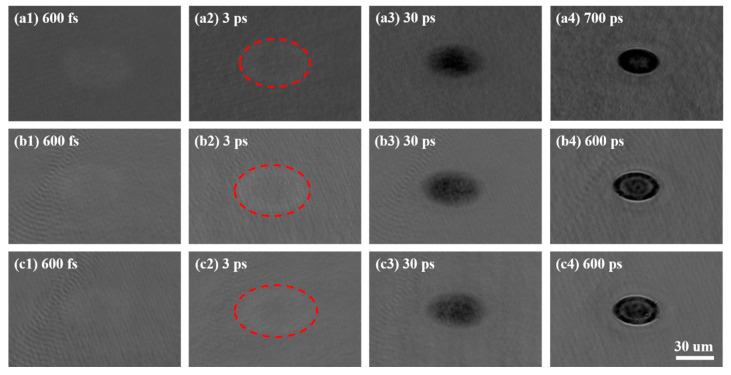
The transient non-equilibrium states of different phase states GST films excited by a single femtosecond laser pulse with the fluence of 0.045 J/cm^2^. (**a1**–**a4**) Images of transient non-equilibrium states of amorphous state films, (**b1**–**b4**) images of transient non-equilibrium states of crystalline FFC structure films, (**c1**–**c4**) images of transient non-equilibrium states of crystalline HCP structure films.

According to the analysis of the XRD patterns, the as-grown, crystalline FCC state, and HCP state films were selected to study the ultrafast dynamics of different phase states, which were the as-deposited films and the films annealed at 250 °C and 350 °C for 30 min, respectively. To obtain a strong signal-to-noise ratio, the laser fluence of 0.045 J/cm^2^ was used to carry out the pump–probe experiments, which is more than the ablation threshold of GST films. The transient non-equilibrium states of different phase states GST films excited by a single femtosecond laser pulse are shown in Figure 4. It can be found that the reflectivity of these films changes with the delay times after the laser irradiation. The areas irradiated by the laser beam of different phase states films are a little obscure and bright at the beginning—sub-picosecond—and form elliptical shapes, because the pump pulse is focused on the sample at an angle of 45°. Then, the center of the areas becomes dark, and the size of the dark regions grows bigger from 3 ps to 30 ps for the three kinds of films. Among different phase states GST films, the excited zone of the crystalline HCP structure sample is the biggest, while that of the amorphous GST is the smallest at the delay time of 30 ps, shown in the red circles in Figure 4. It can be indicated the electron in crystalline HCP structure GST can be excited the most easily, while that in the amorphous state is excited a little more difficultly, which will be studied in the later analysis. As the delay time increases, the center of the area is lighter than the surrounding. The Newton rings emerge at the delay times of 700 ps, 600 ps, and 600 ps for the amorphous, crystalline FCC state, and HCP state films, respectively, which is caused by the constructive and destructive interference of the light reflected from the surface with the light reflected from the layer interface, induced by the formation of a thin layer on the surface as induced by the laser beam [34]. In addition, the background intensities of the shadowgraph images for the amorphous GST films are different from those for the crystalline FCC state and HCP state films, while the background intensities of the images are almost the same for the crystalline FCC state and HCP state films.

**Figure 5:**
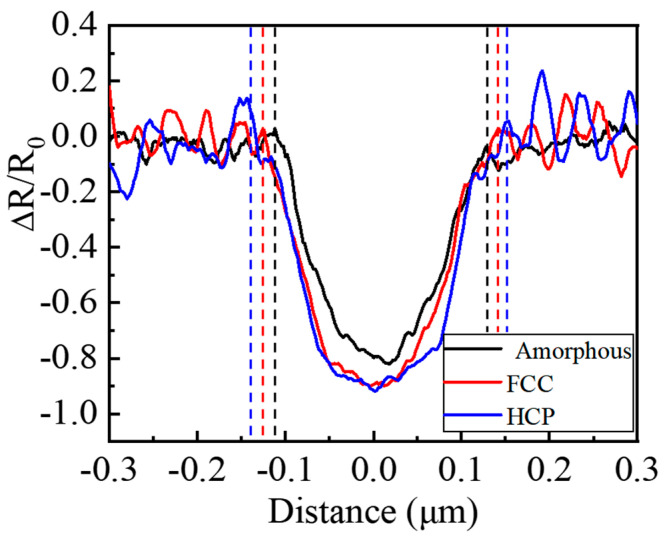
The extracted curve of the spatial reflectivity distributions at the short axis of the irradiated elliptical focal area at the delay time of 100 ps.

Because the original reflectivity *R*_0_ is different for every phase state, which is related to the property of materials, the reflectivity is normalized by *R*_0_ in pump–probe experiments based on Equation (1), and it is meaningful to compare the reflectivity of different materials in the same figure. Thus, the spatial reflectivity distributions of different phase states GST films are extracted from the short axis of the irradiated elliptical focal areas in the images taken in the pump–probe experiments at the delay time of 100 ps, and smoothed using the Savitzky–Golay method, as shown in Figure 5. It can be found that the curves of these films present Gaussian distributions, which are in agreement with the property of the laser beam. The minimum normalized reflectivity of the crystalline HCP structure films is a little smaller than that of the crystalline FCC structure films, while both of them are smaller than that of the amorphous GST films. It can be indicated that the laser energy absorbed by the crystalline HCP structure films is the most, and that of the amorphous GST films is the least among these films. For the short axis radius of the irradiated areas, that of the crystalline HCP structure films is the biggest, and that of the amorphous GST films is the smallest, which can be seen from the vertical lines in Figure 5. It can be inferred that the ablation threshold of the crystalline HCP structure films is the lowest, and that of the amorphous state films is the highest.

**Figure 6:**
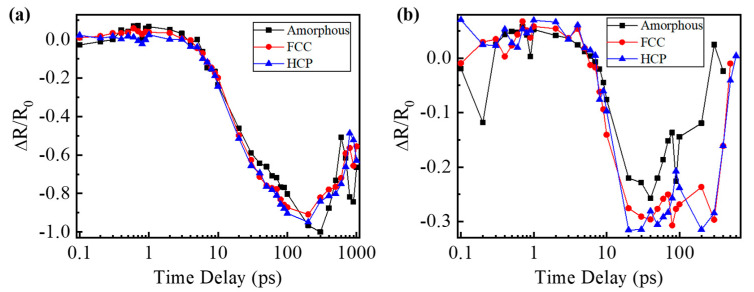
(**a**) Reflectivity dynamics of the zone irradiated by the relatively high laser fluence for different phase states films, (**b**) reflectivity dynamics of the zone irradiated by the relatively low laser fluence for different phase states films.

Because the laser fluence used in the pump–probe experiments is more than the ablation threshold of these films, and the intensity of the laser beam yields a Gaussian distribution, the normalized reflectivity can be divided into two zones to investigate the ultrafast dynamics of different phase states GST films. One zone is irradiated by the relatively high fluence of a laser pulse, and the center of the elliptical area is selected to present the reflectivity of this zone. The other zone is excited by the relatively low fluence of a laser pulse. The position of this zone changes with the delay time at the beginning when the laser beam irradiates the material, and it is the surrounding of the excited region on the films. The normalized reflectivity of two zones is calculated based on Equation (1) and shown in Figure 6. The reflectivity of the area irradiated by a relatively high laser fluence experiences an increase when excited, and there is a maximum value at the delay time of about 600 fs for these films. The reflectivity of amorphous GST films rises the fastest, while that of the crystalline HCP structure films grows the most slowly during this period, as shown in Figure 6a. Although the reflectivity of the area irradiated by a relatively low laser fluence also increases at first after being excited, the curves reach the maximum at around 1 ps, as shown in Figure 6b. The maximum reflectivity is in the curve of the crystalline HCP structure films, while the minimum is in the curve of the amorphous films excited by a relatively low laser fluence. This is because when the femtosecond laser is focused on the GST films, the free carrier is excited first, and then the energy of the laser transfers from the electron to the lattice. Due to annealing, the structure of bonding changes for different phase states [35], and the band gap of the GST films decreases [30]. When a laser pulse with a relatively low fluence is focused on the samples, the electron of the crystalline HCP structure films with a relatively low band gap is easy to excite. Thus, the reflectivity of the irradiated area increases fast, and there is a maximum reflectivity for the crystalline HCP structure films among these samples excited by the same fluence of the laser beam. However, the electron of the amorphous GST is harder to excite due to the relatively high band gap for different films. Similarly, the reflectivity grows slowly, and there is a minimum value among these samples irradiated by the same laser fluence, as shown in Figure 6b, though there are some fluctuations due to the low signal-to-noise ratio. This is why the size of excited zones for the crystalline HCP structure films are the biggest in the circle in Figure 4. As the fluence of the laser pulse increases, the electron of the crystalline HCP structure films with a low band gap will be excited faster and faster. Therefore, the maximum reflectivity decreases, and there is a minimum reflectivity for the crystalline HCP structure films among these samples. Likewise, there is a maximum reflectivity for the amorphous state films with a relatively high band gap among these films, as shown in Figure 6a. Because of the decrease in the band gap after GST films annealed, the electron becomes easier to be excited by the femtosecond laser. When the intensity of the electron reaches the threshold, the films will be ablated [36]. Thus, the ablation threshold of the crystalline HCP structure films is the lowest, and that of the amorphous GST films is the biggest among these samples. It is consistent with the results above. When the delay time increases from ~600 fs to ~200 ps, the reflectivity of the center of the irradiated areas decreases for all the films. That of the crystalline HCP structure films drops a little faster than that of the crystalline FCC structure films, and that of the amorphous GST films declines the most slowly among them. This process represents the energy transfers from the electron to the lattice of the films. This causes a temperature increase in the lattice, which can result in crystallization, and even ablation of the material. When the delay time continues increasing, the reflectivity of the zones irradiated by the relatively high laser fluence returns to growing, then fluctuates due to melting and ablation for all the samples as shown in Figure 6a. Likewise, when a relatively low fluence of laser pulse irradiates the films, the reflectivity falls from the delay time of ~1 ps to ~10 ps. After that, it fluctuates, and this is probably because of the heat transfer from the center, which is irradiated by a relatively high laser fluence, resulting in a temperature difference. 

### 3.3. Crystallization Characterization on Irradiation Areas for Different Phase States Films

**Figure 7:**
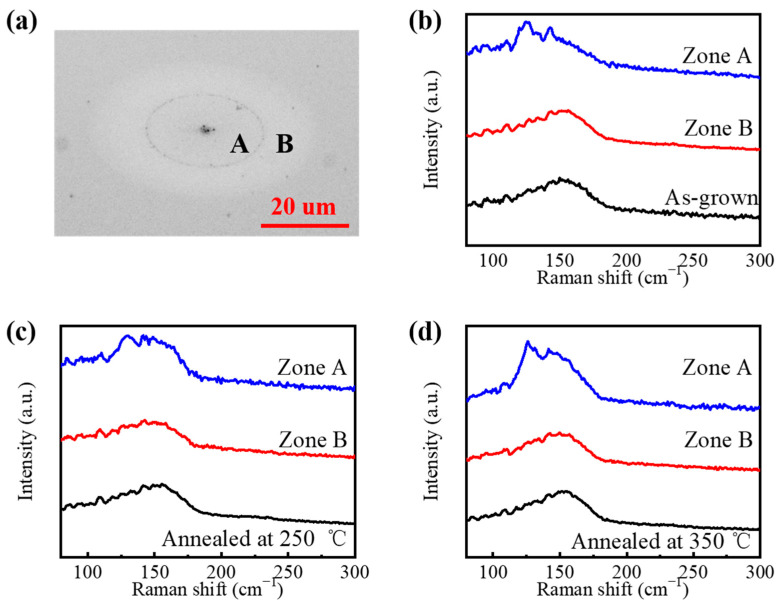
(**a**) Optical images of the final structures of the crystalline FCC structure films after the reflective-type pump–probe experiments, (**b**) Raman spectrum of the amorphous GST films in different regions, (**c**) Raman spectrum of the crystalline FCC structure films in different regions, (**d**) Raman spectrum of the crystalline HCP structure films in different regions.

The crystallization evolution induced by a femtosecond laser beam pulse for different phase state GST films are also investigated on two zones. The final structure of crystalline FCC structure film after pump–probe experiments, as an example, was measured using an optical microscope (Olympus Inc., BX51, Melville, NY, USA), and the optical images are shown in Figure 7a. Zone A is irradiated by the relatively high laser fluence in the center of the structure, where the materials are removed by the laser beam, and a dent is formed. However, the films are thick enough for the laser beam to ablate, and there are still GST materials in the dent. Zone B is irradiated by the relatively low laser fluence at the surrounding of the structure, where the reflectivity changes compared with that of the original films. The Raman spectrum of the original films, Zone A, and Zone B of different phase states films are shown in Figure 7b–d. After the laser beam irradiation, the relative intensity of Peak A to Peak B drops from about 1.4 to about 1.2 in the Raman spectrum of Zone B on the amorphous GST films, and it continues falling from about 1.25 to around 1.15 in Zone B on the crystalline FCC structure films. It is indicated that an amorphous GST film can be crystallized to the FCC structure by a femtosecond laser pulse. This is because the electron will be excited by a femtosecond laser, and have an influence on the atomic diffusion, which will vibrate the covalently bonded backbone of the crystalline lattice and result in crystallization. It is an almost non-thermal phase transition. However, it is hard to control the laser fluence to make the amorphous GST transit to the crystalline HCP structure by a femtosecond laser pulse instead of ablation, because it needs heat accumulation. When a crystalline FCC structure film continues to be irradiated by a femtosecond laser pulse, the crystallization will be enhanced, instead of amorphization. However, it is still hard for it to become the HCP structure. Because the ablation threshold decreases as the crystallization enhances, the laser fluence needs to be controlled more precisely to enhance crystallization. Therefore, the phase transition from the amorphous to the HCP structure can be realized by multiple femtosecond laser pulses with a relatively low fluence to accumulate heat. For a crystalline HCP structure film, there is a shift from Peak C to Peak A after irradiation in Zone B, and the relative intensity of Peak A to Peak B is about 1.42, which is similar to the Raman spectrum of the amorphous GST films. It is indicated that the phase transition to the amorphous state occurs, and it is easy to make a crystalline FCC structure amorphization by a femtosecond laser pulse with the fluence lower than the ablation threshold. It is also because the non-thermal phase change results from the excited electron, which makes the atom freeze in the place to cause amorphization. As for Zone A, the peaks at about 125 cm^−1^ and 143 cm^−1^ appear for all films, which are shown as over-melted states [37].

## 4. Conclusions

In this paper, the ultrafast dynamics of amorphous, crystalline FCC, and HCP structures GST films were studied using reflective-type pump–probe experiments. The variations of normalized reflectivity after a femtosecond laser pulse irradiation show that the electron could be excited more easily and the energy of the laser could be absorbed faster as the annealing temperature increased for GST. This was because the structure of bonding changed for different phase states and the band gap of the GST films decreased after annealing. The amorphous and crystalline FCC structures of GST could be transited to crystalline HCP structures using femtosecond laser pulses, and the crystalline HCP structures of GST could be directly and easily changed to the amorphous state by a femtosecond laser pulse because of the non-thermal phase change resulting from the excited electron. When the crystalline FCC structure film was irradiated by a femtosecond laser pulse, the crystallization was enhanced, instead of amorphization.
